# Has influenza B/Yamagata become extinct and what implications might this have for quadrivalent influenza vaccines?

**DOI:** 10.2807/1560-7917.ES.2022.27.39.2200753

**Published:** 2022-09-29

**Authors:** John Paget, Saverio Caini, Marco Del Riccio, Willemijn van Waarden, Adam Meijer

**Affiliations:** 1 Netherlands Institute for Health Services Research (Nivel), Utrecht, the Netherlands; 2 University of Florence, Florence, Italy; 3 Centre for Infectious Disease Control, National Institute for Public Health and the Environment (RIVM), Bilthoven, the Netherlands

**Keywords:** Influenza, Epidemiology, Virology, Vaccination, WHO

## Abstract

While two influenza B virus lineages have co-circulated, B/Yamagata-lineage circulation has not been confirmed since March 2020. The WHO FluNet database indicates that B/Yamagata-lineage detections were reported in 2021 and 2022. However, detections can result from use of quadrivalent live-attenuated vaccines. Of the type B viruses detected post-March 2020, all ascribed to a lineage have been B/Victoria-lineage. There is need for a global effort to detect and lineage-ascribe type B influenza viruses, to assess if B/Yamagata-lineage viruses have become extinct.

Following the start of the coronavirus (COVID-19) pandemic at the start of 2020, there were important changes in the circulation of influenza viruses around the world and this led to the possible elimination of the influenza B/Yamagata lineage (it had not been conclusively detected since April 2020) [1]. This observation was based on an analysis of sequences from the Global Initiative on Sharing All Influenza Data (GISAID), an influenza virus nucleic acid sequence database, and of World Health Organization (WHO) FluNet data on global influenza virus detection until 1 August 2021 [1]. 

In this rapid communication, we provide an update of the GISAID and FluNet analyses, with a focus on influenza B viruses and B/Yamagata specifically. The analysis includes additional data up to 31 August 2022, which covers the northern hemisphere 2021/22 winter and the start of the 2022 influenza epidemic season in the southern hemisphere and we discuss implications of the possible elimination of the influenza B/Yamagata lineage for surveillance, laboratory diagnostics and influenza vaccination composition. 

## Virological surveillance data

We downloaded the FluNet and severe acute respiratory syndrome coronavirus 2 (SARS-CoV-2) databases from 1 January 2017 to the end of August 2022 [2,3]. The Figure shows that influenza activity was greatly reduced following the emergence of SARS-CoV-2 but there was an important increase in influenza activity from the autumn of 2021 onwards. Table 1 outlines sequences of the B/Yamagata haemagglutinin (HA) genome segment uploaded to the GISAID database from 2017 through August 2022 [4]. This table confirms the decline in B/Yamagata sequences that started in 2019 (both in terms of number of sequences and number of countries reporting) and that no influenza B/Yamagata sequences have been uploaded with specimen collection dates after March 2020 [1,5].

**Figure:**
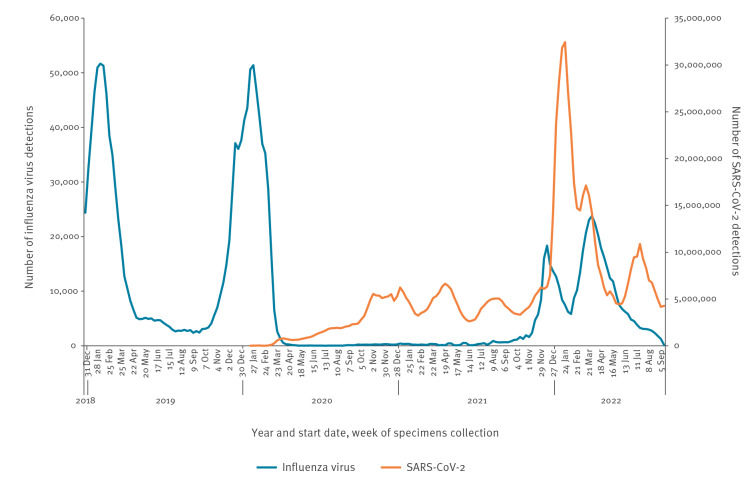
Influenza and SARS-CoV-2 detections by year and week, World Health Organization global surveillance data, 1 January 2019–August 2022

SARS-CoV-2: severe acute respiratory syndrome coronavirus 2.

Sources: FluNet [2], COVID-19 dashboard [3].

**Table 1:** Influenza B/Yamagata virus haemagglutinin segment sequences by year of specimen collection, GISAID database, 1 January 2017–29 August 2022 (n = 12,559)

Year	B/Yamagata	Number of countries	Countries (number of sequences)
2017	4,888	121	Too many to list
2018	6,564	133	Too many to list
2019	988	74	Too many to list
2020	119	14	United States (75), France (10), Tunisia (8), Norway (6), Russia (5), Chile (4), Trinidad and Tobago (3), Australia (2), Germany (1), Honduras (1), Jamaica (1), Japan (1), South Korea (1), Spain (1)
2021	0	0	NA
2022	0	0	NA

NA: not applicable.

Source: GISAID; accessed on 29 August 2022. GISAID also captures sequences from other public sequence databases (e.g. GenBank) and since the lineage for the type B influenza virus sequences captured by GISAID in this way is frequently unknown, we downloaded the haemagglutinin sequences of these viruses, phylogenetically assigned them to B/Victoria or B/Yamagata lineage and added these to the overall analysis. We thank GISAID for access to their database and all submitters of data and sequences to the database (Supplement).

Table 2 outlines the influenza A and B data in the FluNet database from 2017 through August 2022. As expected, influenza B represented 9–40% of detections [6], but there has been a major decline in influenza B/Yamagata cases since 2019, with only 43 detections reported in 2021 (mostly from China) and sporadic detections (n = 8) reported in 2022. The last reported detections of B/Yamagata were in Pakistan (n = 4) and Belize (n = 1) in week 22 (31 May–5 June) of 2022. Importantly, the percentage of influenza B detections where the lineage was determined increased dramatically over this period, from 30% in 2017 to 84% in 2022, and this corroborates efforts made by the WHO Global Influenza Surveillance and Response System (GISRS) National Influenza Centres (NICs) to perform a comprehensive surveillance of influenza B lineages [7,8].

**Table 2:** World Health Organization FluNet surveillance data for influenza by year of specimen collection, 1 January 2017–5 September 2022

Year	Total influenza	Influenza A	%	Influenza B	%	Total influenza B detections						% influenza B detections (excl. lineage not determined)	
						Lineage not determined	%	B/Victoria	%	B/Yamagata	%	B/Victoria	B/Yamagata
2017	588,291	444,991	75.6	143,300	24.4	100,354	70.0	12,394	8.6	30,552	21.3	28.9	71.1
2018	739,833	443,908	60.0	295,925	40.0	238,164	80.5	6,237	2.1	51,524	17.4	10.8	89.2
2019	789,011	657,369	83.3	131,642	16.7	82,935	63.0	45,243	34.4	3,464	2.6	92.9	7.1
2020	471,106	303,038	64.3	168,068	35.7	142,184	84.6	25,520	15.2	364	0.2	98.6	1.4
2021	116,225	82,181	70.7	34,044	29.3	4,898	14.4	29,103	85.5	43; China (32), India (3), United States (2), Mexico (1), Niger (1), Afghanistan (1), Ghana (1), Ivory Coast (1), Bulgaria (1)	0.1	99.9	0.1
2022	363,251	329,688	90.8	33,563	9.2	5,150	15.3	28,405	84.6	8; Chad (1; week 3), Germany (1; week 12 and 1; week 13), Pakistan (4, week 22), Belize (1; week 22)	0.0	100.0	0.0

We thank FluNet for access to their database and all submitters of data to this database.

China reported a large influenza B/Victoria epidemic in 2020 (end of the year) and 2021 (99.8% of cases were B/Victoria), but there were also 83 B/Yamagata detections reported [9]. Importantly, a number of the B/Yamagata detections were reported to the FluNet database (China reported the most cases of B/Yamagata in 2021 and 2022 (32/48 cases; 67%); Table 2), but there were no sequences uploaded to GISAID (Table 1).

## Discussion

Seasonal influenza epidemics are caused by influenza A and B virus types and a study covering 29 countries worldwide found that the median proportion of influenza cases caused by influenza B virus was 23% [6,10]. Influenza B was the dominant virus type in about one in every seven seasons [6]. Importantly, two antigenically distinct B phylogenetic lineages, B/Victoria/2/87-lineage (B/Victoria) and B/Yamagata/16/88-lineage (B/Yamagata), emerged in the 1970s [11] and have co-circulated globally at least since 2001 [11,12]. 

Until 2012, influenza vaccinations were based on a trivalent vaccine composition, with one influenza A(H3) strain, one influenza A(H1) or A(H1)pdm09 (since 2010) strain and one influenza B strain [13]. The unpredictable circulation of influenza B/Victoria and B/Yamagata lineages made the strain selection for influenza vaccines difficult and influenza B lineage mismatches were common (more than 40% of seasons in temperate countries and 30% in tropical areas) [6]. Furthermore, both lineages had become antigenically distinct, not offering any cross-protection [11,14]. In a push to address this problem, quadrivalent influenza vaccines (QIV) were introduced in February 2012 which included both an influenza B/Yamagata and a B/Victoria strain [13] and were approved by the European Medicines Agency in 2013 [15]. The disappearance of B/Yamagata would potentially have important implications for the use of QIV, which are now common in Europe. Possible scenarios include the continuation of the QIV with a new strain (e.g. an additional influenza H3, H1 or B/Victoria strain), which might help reduce vaccine mismatches, or a return to trivalent influenza vaccines.

Our analysis of the GISAID database confirms the findings and conclusions of Dhanasekaran et al. with 13 months of additional data [1]. However, the FluNet database showed that while influenza B/Yamagata largely disappeared in 2021 and especially 2022, one is still seeing sporadic detections of the virus and these occurred in very diverse regions of the world in 2022 (Table 2).

The B/Yamagata detection results from FluNet are important but caution is currently needed in their interpretation as the sporadic B/Yamagata detections could be vaccine-derived. They could be linked to the presence of live-attenuated influenza vaccine (LAIV) preparations in the environment or RNA from traditional inactivated vaccines if they were administered in the same room where specimens are also collected from patients [16,17]. This probably means that each current detection of influenza B/Yamagata reported to FluNet cannot be taken as a true circulation of wild-type B/Yamagata virus without further, and detailed, virological (sequence confirmation) and epidemiological (i.e. vaccination status, timing of vaccination and vaccine type) data. Indeed, a number of B/Yamagata detections in Scotland and the United States during the 2021/22 season were confirmed as being linked to LAIV [18,19]. Another important point is that if the B/Yamagata lineage does become extinct, there is the potential of a later re-introduction of this influenza virus lineage (as has happened in the past, e.g. the re-emergence of influenza A(H1N1) in 1977) and this lineage could still pose a risk in the coming years [1].

It is also of note that the HA genome segment sequence of none of the B/Yamagata detections reported to FluNet in 2021 and 2022 was uploaded to the GISAID sequence database. With regard to this point, our analysis suggests two critical points: (i) that the lineage of a substantial proportion of type B influenza virus detections is not determined and (ii) that only a small proportion of detected B/Yamagata virus sequences are uploaded to GISAID (e.g. in 2020, FluNet had ca 170,000 influenza B virus detections, of which only 15.4% were lineage-determined and 364 were B/Yamagata detections, while GISAID only had 119 (33%) HA genome segment sequences).

To determine extinction of B/Yamagata it is critical that all influenza B viruses are lineage-determined and especially those identified as B/Yamagata are confirmed by sequencing. This appears to be a challenge. The small proportion of sequences of detected B/Yamagata viruses that are uploaded to GISAID might be the result of FluNet being a routine surveillance database that aims to capture all cases in a country through virus detections (for which the lineage of B viruses is not necessarily determined), while the GISAID database represents, for many countries, only a sample of influenza virus-positive specimens that are collected and successfully sequenced by NICs and other specialised laboratories and uploaded for public access. This leaves a considerable proportion of detected B/Yamagata viruses uncharacterised and hampers the assessment of the possible extinction of B/Yamagata. 

An explanation for the lack of B/Yamagata sequences might be that these detections were vaccine-derived, which often only result in very low virus loads in the clinical specimens. This means that even though the NICs have a general policy to sequence all low-prevalent virus subtypes/lineages, it is possible that due to the low viral load, none of the sequenced B/Yamagata-positive specimens resulted in high-quality sequences that could be uploaded to GISAID. Another explanation is that the sequences were not uploaded because they were LAIV-derived [18,19] and thus not reflecting true circulation of B/Yamagata viruses. 

From a laboratory perspective, we think it would be advisable to increase the capability and capacity to determine the lineage of all detected influenza B viruses around the world as this is critical to determine the absence of B/Yamagata lineage viruses. As by our knowledge, no commercial RT-PCR kits for B lineage determination exist; this could be achieved by simple in-house-developed lineage-specific multiplex RT-PCR [20] that can be used in primary diagnostics or with the CDC Atlanta provided kit for public health laboratories [7,8,21]. Furthermore, primary diagnostic laboratories are strongly advised to forward each detected influenza B virus to their NIC for further investigation.

## Conclusion

Influenza B/Yamagata is being closely monitored by WHO GISRS NICs and the WHO Collaborating Centres for influenza. We think it would be advisable to also establish a dedicated multi-disciplinary working group (virologists, immunologists, epidemiologists, modellers, policymakers, experts from GISRS and industry), under the auspices of the WHO, that publishes and communicates criteria to define the moment when B/Yamagata is declared extinct (e.g. 24 months of no new wild-type cases), the data that should be collected (e.g. epidemiological data to define wild-type cases) and the consequences of this declaration (e.g. vaccine composition).

## Supplementary Data

42000 Supplement
